# Administration of Downstream ApoE Attenuates the Adverse Effect of Brain ABCA1 Deficiency on Stroke

**DOI:** 10.3390/ijms19113368

**Published:** 2018-10-28

**Authors:** Xiaohui Wang, Rongwen Li, Alex Zacharek, Julie Landschoot-Ward, Fengjie Wang, Kuan-Han Hank Wu, Michael Chopp, Jieli Chen, Xu Cui

**Affiliations:** 1 Department of Neurology, Henry Ford Hospital, Detroit, MI 48202, USA; 163.wangxh@163.com (X.W.); rli3@hfhs.org (R.L.); Alexzacharek@gmail.com (A.Z.); JLandsc@hfhs.org (J.L.-W.); fwang2@hfhs.org (F.W.); mchopp1@hfhs.org (M.C.); jchen4@hfhs.org (J.C.); 2 Department of Physics, Oakland University, Rochester, MI 48309, USA; kwu1@hfhs.org; 3 Department of Public Health Sciences, Henry Ford Hospital, Detroit, MI 48202, USA

**Keywords:** ATP-binding cassette transporter A1, apolipoprotein E, apolipoprotein E receptor 2, high density lipoprotein, motor function, stroke

## Abstract

The ATP-binding cassette transporter member A1 (ABCA1) and apolipoprotein E (ApoE) are major cholesterol transporters that play important roles in cholesterol homeostasis in the brain. Previous research demonstrated that specific deletion of brain-ABCA1 (ABCA1^−B/−B^) reduced brain grey matter (GM) and white matter (WM) density in the ischemic brain and decreased functional outcomes after stroke. However, the downstream molecular mechanism underlying brain ABCA1-deficiency-induced deficits after stroke is not fully understood. Adult male ABCA1^−B/−B^ and ABCA1-floxed control mice were subjected to distal middle-cerebral artery occlusion and were intraventricularly infused with artificial mouse cerebrospinal fluid as vehicle control or recombinant human ApoE2 into the ischemic brain starting 24 h after stroke for 14 days. The ApoE/apolipoprotein E receptor 2 (ApoER2)/high-density lipoprotein (HDL) levels and GM/WM remodeling and functional outcome were measured. Although ApoE2 increased brain ApoE/HDL levels and GM/WM density, negligible functional improvement was observed in ABCA1-floxed-stroke mice. ApoE2-administered ABCA1^−B/−B^ stroke mice exhibited elevated levels of brain ApoE/ApoER2/HDL, increased GM/WM density, and neurogenesis in both the ischemic ipsilateral and contralateral brain, as well as improved neurological function compared with the vehicle-control ABCA1^−B/−B^ stroke mice 14 days after stroke. Ischemic lesion volume was not significantly different between the two groups. In vitro supplementation of ApoE2 into primary cortical neurons and primary oligodendrocyte-progenitor cells (OPCs) significantly increased ApoER2 expression and enhanced cholesterol uptake. ApoE2 promoted neurite outgrowth after oxygen-glucose deprivation and axonal outgrowth of neurons, and increased proliferation/survival of OPCs derived from ABCA1^−B/−B^ mice. Our data indicate that administration of ApoE2 minimizes the adverse effects of ABCA1 deficiency after stroke, at least partially by promoting cholesterol traffic/redistribution and GM/WM remodeling via increasing the ApoE/HDL/ApoER2 signaling pathway.

## 1. Introduction

Stroke is the leading cause of death and physiological disability worldwide due to both grey matter (GM) and white matter (WM) damage after cerebral ischemic and hemorrhagic injury [1]. There is a need to investigate the molecular mechanisms of strokes, to develop therapeutic strategies to improve GM/WM remodeling, and to improve functional outcome after stroke.

Cholesterol is a major component of myelin and neuronal membranes, and participates in myelination and neuronal function [2]. Thus, cholesterol metabolism, synthesis, and transport substantially impacts GM/WM remodeling and neurological functional recovery after brain injury [3,4,5]. In adult brains, the ATP-binding cassette transporter member A1 (ABCA1) and apolipoprotein-E (ApoE) are major cholesterol transporters involved in the maintenance of cholesterol homeostasis. ABCA1 regulates the efflux of intracellular cholesterol and phospholipids mainly from astrocytes, which form the extracellular ApoE-phospholipid-cholesterol complex. ApoE, the major apolipoprotein in brain, transports lipids and cholesterol particles back into neurons via ApoE receptors (ApoERs) [6,7,8,9]. Therefore, the ApoE/ApoER pathway plays an important role in regulating cholesterol and lipid clearance, transport, and distribution in the brain [10,11,12,13]. Specific brain-ABCA1 knockout (ABCA1^−B/−B^) mice are generated using ABCA1-floxed (ABCA1^fl/fl^) mice with nestin-cre mice [14]. This leads to an absence of ABCA1 in all nestin-lineage cells (neural stem cells), including neurons and glia [14]. Our previous study and other people’s studies show that ABCA1^−B/−B^ mice exhibit reduced ApoE levels in brain tissue (40–50% reduction) and low levels of high density lipoprotein (HDL) in the cerebrospinal fluid (CSF) [14,15,16,17], which shows that ABCA1 regulates ApoE. These mice also have decreased GM/WM densities in the ischemic brain and decreased neurological functional outcomes after stroke injury [16,17]. However, to the best of our knowledge, there are no reports on whether and how ApoE affects cholesterol traffic or redistribution in the brain or in cultured cells, and whether administration of downstream ApoE minimizes the adverse effects of ABCA1 deficiency.

ApoE is a 34 kDa glycoprotein mainly synthesized and released from astrocytes in the brain. ApoE occurs in three major common isoforms in humans: ApoE2, ApoE3, and ApoE4. There is increasing evidence that ApoE2, but not ApoE3 or ApoE4, is associated with dendritic remodeling and synaptogenesis in vivo and in vitro [18,19,20,21,22,23]. In this study, in order to investigate whether administration of cerebral ApoE2 would compensate for the loss of brain ABCA1 after stroke via increasing ApoER2, ABCA1^fl/fl^ and ABCA1^−B/−B^ mice, primary cultured neurons (PCNs), and oligodendrocyte progenitor cells (OPCs) were used.

## 2. Results

### 2.1. ApoE2 Increases ApoE/HDL Level in the CSF and Improves Functional Outcome in ABCA1^−B/−B^-Stroke Mice

**Figure 1:**
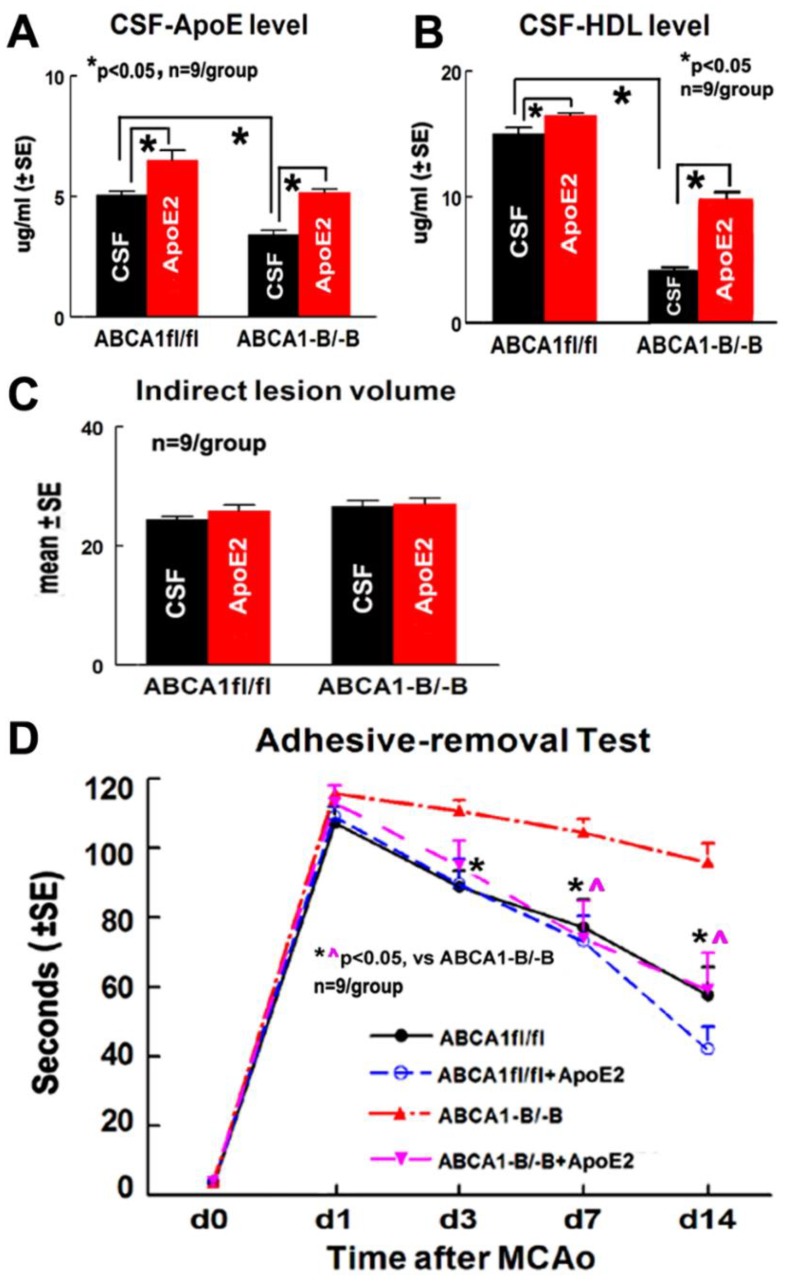
Administration of apolipoprotein E2 (ApoE2) increased the levels of apolipoprotein E (ApoE) and high density lipoprotein (HDL) in the cerebrospinal fluid (CSF) of both ABCA1-floxed (ABCA1^fl/fl^) and specific brain-ABCA1 knockout (ABCA1^−B/−B^) stroke mice, and improved functional outcome in ABCA1^−B/−B^, but not in ABCA1^fl/fl^ stroke mice 14 days after distal middle-cerebral artery occlusion (dMCAo). (**A**) ApoE level in the CSF of mice, (**B**) HDL level in the CSF of mice, (**C**) lesion volume measurement data, and (**D**) the adhesive removal test.

Both the ABCA1^fl/fl^ stroke mice administered with ApoE2 and ABCA1^−B/−B^ stroke mice administered with ApoE2 exhibited significantly increased ApoE (Figure 1A) and HDL (Figure 1B) levels in the CSF 14 days after distal middle-cerebral artery occlusion (dMCAo) (*p* < 0.05, *n* = 9/group). No statistically significant differences were observed in the indirect lesion volume 14 days after stroke between these two groups (Figure 1C).

There was no baseline (i.e., in the absence of CSF and ApoE2) difference at day one among the four groups for the adhesive removal tests, which indicates a balance of wild-type and gene-knockout, and ApoE2 treatment and vehicle-control populations. However, a significant (*p* = 0.0039) interaction effect was detected, which indicates that the functional outcome with gene/treatment effect varies with time. Further analysis indicated that ABCA1^−B/−B^ stroke mice exhibited decreased functional outcome compared with ABCA1^fl/fl^ stroke mice 3, 7, and 14 days after dMCAo. ABCA1^−B/−B^ stroke mice administered ApoE2 showed significantly improved functional outcome at 7 and 14 days after dMCAo compared with CSF administered mice (Figure 1D, *p* < 0.05, *n* = 9/group). However, a negligible functional effect was observed in ApoE2 administered ABCA1^fl/fl^ stroke mice compared to CSF administered ABCA1^fl/fl^ stroke mice.

### 2.2. ApoE2 Increases GM and WM Densities in ABCA1^−B/−B^-Stroke Mice

**Figure 2:**
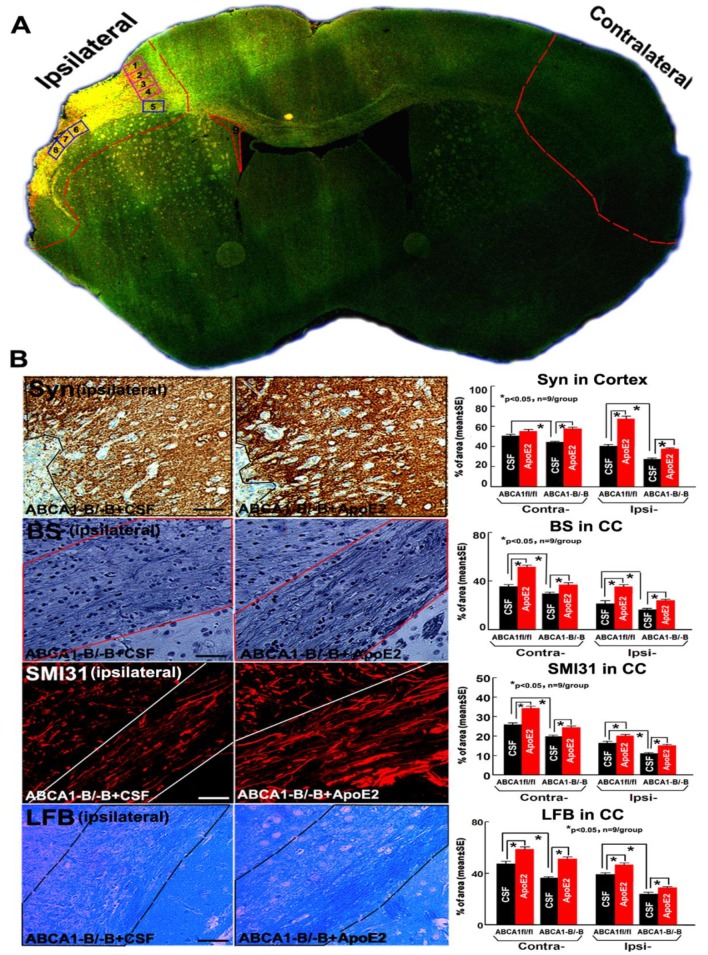
Administration of ApoE2 increased brain grey matter (GM) and white matter (WM) densities in ABCA1^−B/−B^ stroke mice 14 days after dMCAo: (**A**) Confocal-micrograph photo schematically shows the areas where the images were taken for Synaptophysin (Syn, square 1–4) and dendrite morphologies (square 2, 3) or Bielschowsky silver (BS)/Luxol Fast Blue (LFB)/phosphorylated high-molecular weight neurofilament (SMI31) (squares 5–8), nestin/Sox2 (square 9) and ischemic ipsilateral tissue and contralateral brain tissue (outlined areas); (**B**) Syn, BS, SMI31, and LFB staining and quantitative data. Scar bar = 40 µm.

To further investigate mechanisms underlying ApoE2-mediated functional improvement, histochemical and immunohisto staining were employed for both ABCA1^fl/fl^ and ABCA1^−B/−B^ brain samples. Administration of ApoE2 in ABCA1^fl/fl^ stroke mice significantly increased Synaptophysin (Syn^+^, a presynaptic protein, employed herein as a marker of GM) density in the cortex in the ipsilateral brain, and increased Bielschowsky silver (BS^+^, an axon marker), phosphorylated high-molecular weight neurofilament (SMI31^+^, a marker of phosphorylated-neurofilament), and Luxol Fast Blue (LFB^+^, a myelin marker) densities in the corpus callosum (CC) in both the contralateral and the ipsilateral brain compared with administration of CFS in mice (Figure 2B, *p* < 0.05, *n* = 9/group).

**Figure 3:**
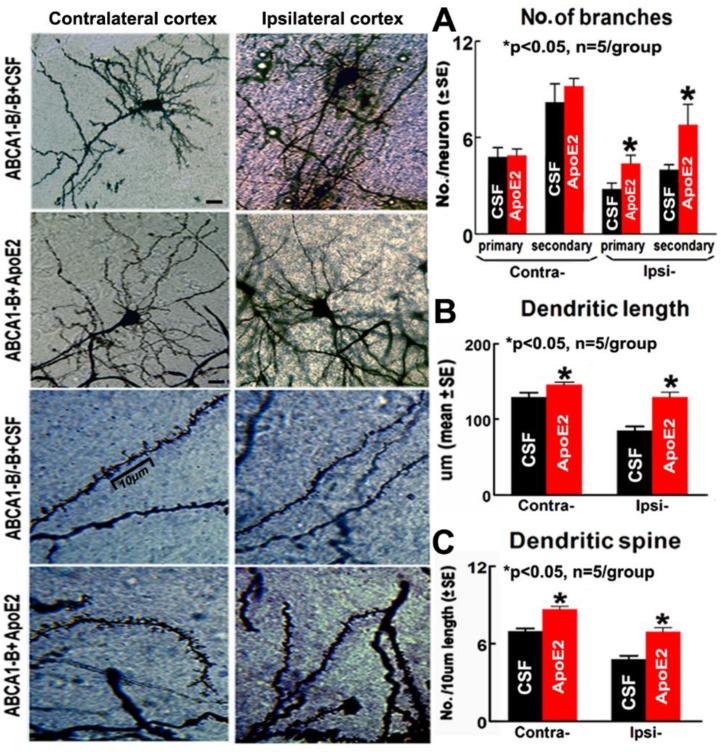
Administration of ApoE2 increased the numbers of dendritic branches, dendritic length, and dendritic spines in the brain of ABCA1^−B/−B^ stroke mice 14 days after dMCAo: (**A**) quantitative data of the neuronal dendritic branch, (**B**) quantitative data of the neuronal dendritic length, and (**C**) measurement data of dendritic spine. Scar bar = 10 µm. * *p* < 0.05, *n* = 5/group.

Compared with ABCA1^−B/−B^ stroke mice administered with CSF, the ABCA1^−B/−B^ stroke mice administered with ApoE2 also exhibited significantly increased Syn^+^, BS^+^, SMI31^+,^ and LFB^+^ densities in both the contralateral and the ipsilateral brain (Figure 2B, *p* < 0.05, *n* = 9/group). In addition, ApoE2 administered ABCA1^−B/−B^ stroke mice exhibited significantly increased numbers of primary/secondary dendritic branches and dendritic spines (particularly the mushroom-shaped mature spines) in the cortex, and dendritic length in the ipsilateral region. Moreover, significant increases in dendritic lengths and dendritic spines were detected in the contralateral region compared with CSF administered ABCA1^−B/−B^ stroke mice (Figure 3, *p* < 0.05, *n* = 5/group).

### 2.3. ApoE2 Increases ApoER2, Synaptic Protein and Myelin Protein Level in the Ischemic Brain

**Figure 4:**
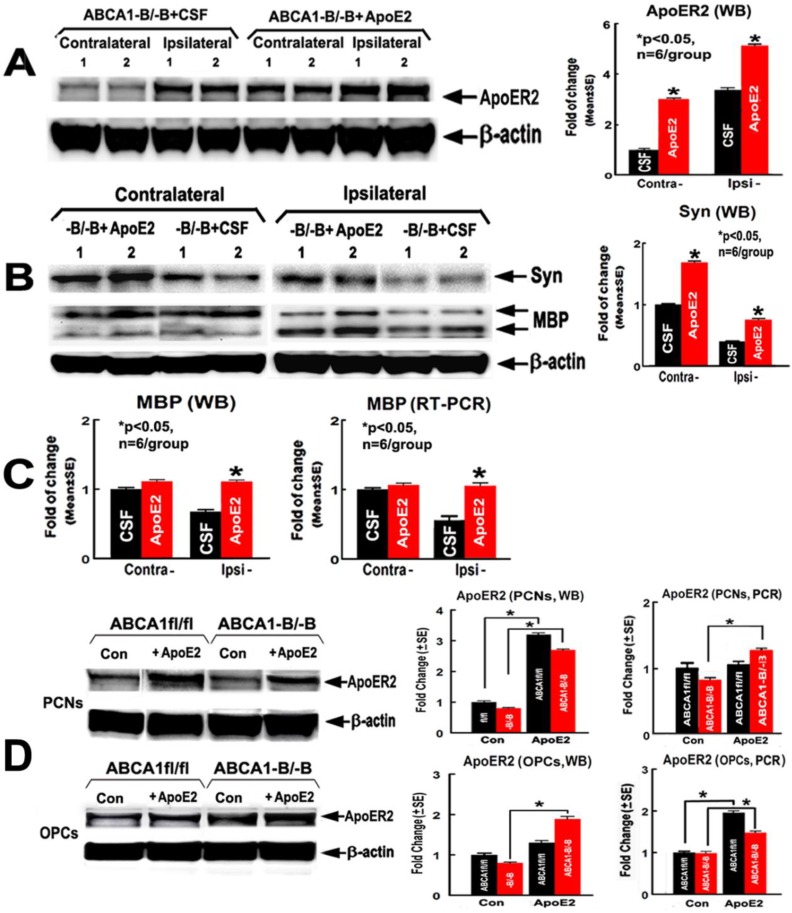
Administration of ApoE2 increased brain ApoER2, Syn, and myelin basic protein (MBP) levels in ABCA1^−B/−B^ stroke mice 14 days after dMCAo. ApoE2 treatment elevated ApoER2 levels in both primary cultured neuron (PCN) and oligodendrocyte-progenitor cell (OPC) cultures. (**A**) ApoER2 western blot (WB) assay and quantitative data in brains; (**B**) Syn WB assay and quantitative data in brains; (**C**) MBP WB and real-time reverse transcription polymerase chain reaction (RT-PCR) quantitative data in brains; (**D**) ApoER2 WB and RT-PCR data in PCN and OPC cultures. * *p* < 0.05, *n* = 6/group.

To test whether administration of ApoE2 increases ApoER2, synaptic protein and myelin protein expression in brains of ABCA1^−B/−B^ stroke mice, the brain protein/mRNA level of ApoER2, Syn, and myelin basic protein (MBP, a myelin marker) were measured. The ApoER2 protein/mRNA levels in both PCN and OPC cultures were evaluated by using western blot (WB) and real-time reverse transcription polymerase chain reaction (RT-PCR) assays. The data show that the protein level of ApoER2 (Figure 4A) and Syn (Figure 4B), in both the contralateral and the ipsilateral brain tissues, and the protein/mRNA levels of MBP (Figure 4C) in the ipsilateral brain, were significantly elevated in ABCA1^−B/−B^ stroke mice treated with ApoE2 compared with ABCA1^−B/−B^ stroke mice treated with CSF (*p* < 0.05, *n* = 6/group). In addition, ApoE2 increased the ApoER2 protein levels in both ABCA1^fl/fl^-PCNs and ABCA1^−B/−B^-PCNs, and increased ApoER2 mRNA level in ABCA1^−B/−B^-PCNs. Administration of ApoE2 significantly increased the ApoER2 protein levels in ABCA1^−B/−B^-OPCs, and increased ApoER2 mRNA level in both ABCA1^fl/fl^-OPCs and ABCA1^−B/−B^-OPCs (Figure 4D, *p* < 0.05, *n* = 6/group).

### 2.4. ApoE2 Enhances Neurogenesis in the Brain of ABCA1^−B/−B^ Stroke Mice

**Figure 5:**
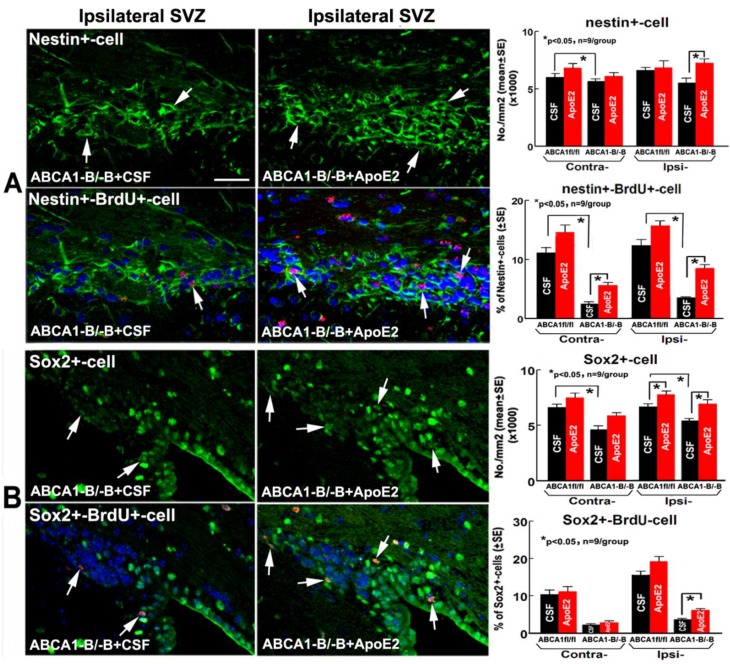
Administration of ApoE2 increased neurogenesis in the brain of ABCA1^−B/−B^ stroke mice 14 days after dMCAo. (**A**) nestin (green) double with or without BrdU (red)/4′,6-diamido-2-phenylindole (DAPI) (blue) immunostaining in both the contralateral and ipsilateral subventricular zone (SVZ), and quantitative data; (**B**) Sox2 (green) double with or without BrdU (red)/DAPI (blue) immunostaining in the contralateral-CC or ipsilateral-CC, and quantitative data. Scar bar = 40 µm, * *p* < 0.05, *n* = 9/group.

Except for the number of Sox2^+^ cells (a marker of proliferating neural progenitor cells-NPCs) in the ipsilateral subventricular zone (SVZ) significantly increasing in ApoE2 administered ABCA1^fl/fl^ stroke mice, no significant deference was found between CSF- and ApoE2-administered ABCA1^fl/fl^ stroke mice. No significant differences were observed between administration of CSF and ApoE2 groups in the number of nestin^+^ cells (a marker of NPCs) and the percentage of Sox2^+^/BrdU^+^ cells (proliferating NPCs) in the contralateral SVZ in ABCA1^−B/−B^ stroke mice. However, the percentage of nestin^+^/BrdU^+^-cells (newly-formed NPCs) and the number of Sox2^+^ cells in the contralateral SVZ significantly increased, and the number of nestin^+^-/Sox2^+^ and nestin^+^-/Sox2^+^ double with BrdU^+^ cells in the ipsilateral SVZ significantly increased in ApoE2 administered ABCA1^−B/−B^ stroke mice compared with CSF administered ABCA1^−B/−B^ stroke mice. These data indicate that intracerebral administration of ApoE2 significantly increases neurogenesis in ABCA1^−B/−B^ stroke mice 14 days after dMCAo (Figure 5, *p* < 0.05, *n* = 9/group).

### 2.5. ApoER2 Co-Localizes in PCNs, and Supplementation with ApoE2 Increases Cholesterol Uptake in PCNs

**Figure 6:**
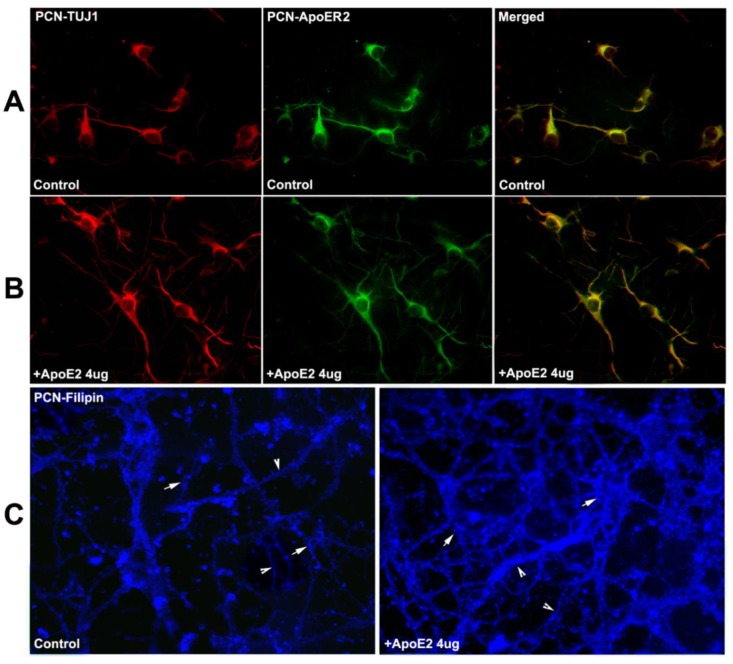
ApoER2 is colocalized with neuron-specific class III-tubulin (TUJ1)^+^-PCNs; ApoE2 treatment increases cholesterol uptake/redistribution in C57BL/6-PCNs. (**A**) PCNs in vehicle-control group; (**B**) PCNs in ApoE2 (4 µg/mL) treatment group; (**C**) Filipin III stained PCNs with or without ApoE2-treatment (arrow points cholesterol particles in soma of PCNs, arrow-head shows cholesterol particles in dendrites of PCNs).

Immuno-fluorescent staining showed that the ApoER2 is co-localized in neuron-specific class III-tubulin (TUJ-1, a phenotypic marker of neural cells) positive neurons, and there are more dendrites with supplementation of ApoE2 (4 µg/mL) PCNs than in the vehicle-control PCNs (Figure 6A,B). Filipin III staining showed that the overall fluorescence or subcellular distribution of the free cholesterol particles both within the soma (arrows) and dendrites (arrow heads) of PCNs significantly increased in the ApoE2 treatment group compared with the vehicle-control group (Figure 6C).

### 2.6. ApoE2 Increases Neurite and Axonal Outgrowth in ABCA1^−B/−B^-PCNs

**Figure 7:**
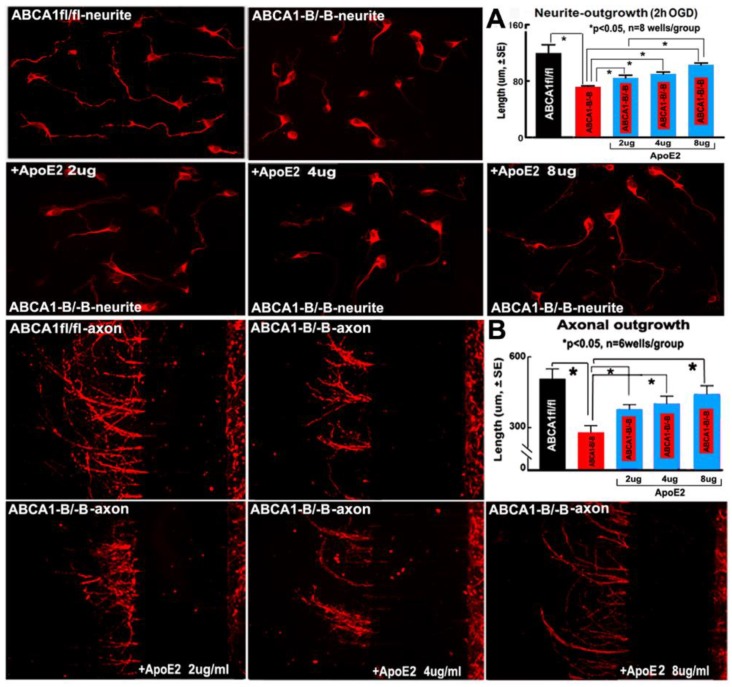
ApoE2 treatment increased neurite and axonal outgrowth in ABCA1^−B/−B^-PCNs. (**A**) neurite outgrowth and measurement data after oxygen-glucose deprivation (OGD); (**B**) axonal outgrowth and measurement data. * *p* < 0.05, *n* = 6 wells/group.

Neurite length after oxygen-glucose deprivation (OGD) (Figure 7A) and the axonal outgrowth (Figure 7B) were significantly reduced in ABCA1^−B/−B^ PCNs compared with ABCA1^fl/fl^ PCNs (*p* < 0.05, *n* = 6 wells/group). However, treatment of ABCA1^−B/−B^ PCNs with ApoE2 at 2, 4, and 8 µg/mL significantly increased neurite and axonal outgrowth compared with non-treatment ABCA1^−B/−B^ PCNs (*p* < 0.05, *n* = 6 wells/group).

### 2.7. ApoE2 Increases Proliferation and Survival in ABCA1^−B/−B^-OPCs 

**Figure 8:**
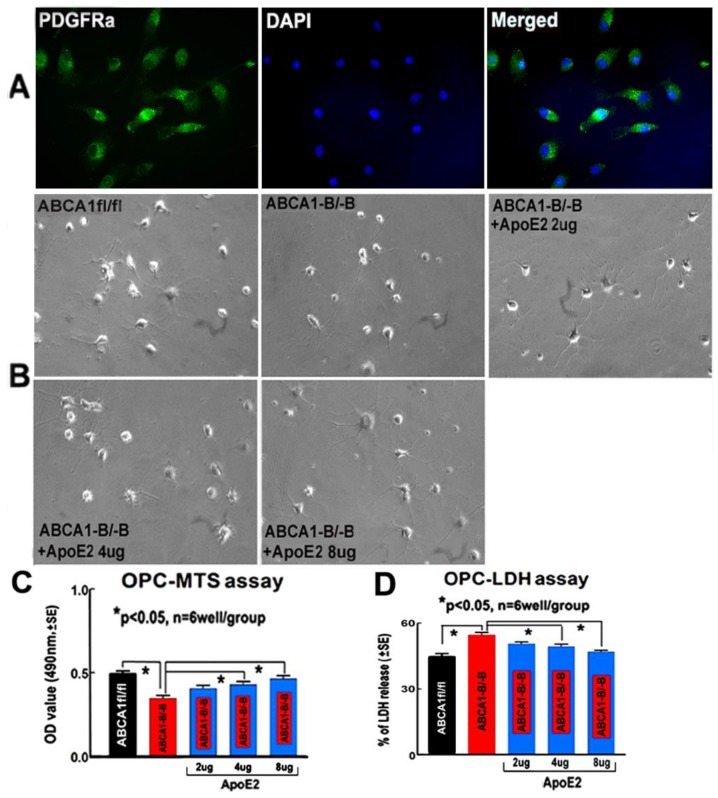
ApoE2 treatment increased OPC proliferation/survival. (**A**) The OPC images immunofluorescent-stained with platelet-derived growth factor receptor alpha (PDGFRa, a marker of OPCs) (green), O4 (red), DAPI (blue), and merged; (**B**) the light images of OPCs and treatment with different doses of ApoE2; (**C**) 3-[4,5-dimethylthiazol-2-yl]-5-[3-carboxymethoxyphenyl]-2-[4-sulfophenyl]-2H-tetrazolium (MTS) assay; and (**D**) the lactate dehydrogenase (LDH) assay. * *p* < 0.05, *n* = 6 wells/group.

From the OPC representative images (Figure 8A,B), and the OPC proliferation (Figure 8C) and death (Figure 8D) measurement data, we found that the proliferation and survival of ABCA1^−B/−B^-OPCs significantly decreased compared with ABCA1^fl/fl^-OPCs. However, supplementation of ABCA1^−B/−B^-OPCs with 4 or 8 µg/mL of ApoE2 significantly minimized the reduction in proliferation/survival in ABCA1^−B/−B^-OPCs (*p* < 0.05, *n* = 6 wells/group), and 2 µg/mL ApoE2 had no obvious benefit compared with control of ABCA1^−B/−B^-OPCs.

## 3. Discussion

Stroke-induced GM/WM damage causes serious long-term neurological deficits, partly due to highly constrained axonal regeneration and remyelination in the adult brain. Injury to WM disrupts axonal connections, results in motor dysfunction, and produces primarily unilateral functional deficits [24]. Focal cerebral ischemia in the adult rodent increases neurogenesis primarily by activation of neural progenitor and stem cells in the SVZ of the lateral ventricle. These augmented and newly-formed NPCs migrate and generate new neurons in the ischemic brain that disperse to the GM/WM [19,25]. Thus, neurogenesis plays an important role in GM/WM remodeling and functional recovery after stroke. ApoE-/- mice display fewer neurons with shorter neurite outgrowth impaired regeneration in the dentate gyrus of the hippocampus, reduced synaptogenesis, and increased cortical lesions than wild-type mice [20,21,26]. Although murine ApoE is monomorphic, the homology of mouse and human ApoE is 70%, and human and mouse ApoE have similar molecular weights (34 vs. 33 kDa, respectively) [27]. In the current study, in order to investigate whether intracerebral administration of ApoE2 promotes GM/WM remodeling, we measured GM/WM densities and neurogenesis. Although administration of ApoE2 increased WM density, negligible neurogenesis was found in ABCA1^fl/fl^ stroke mice. However, intracerebral infusion of ApoE2 significantly increased GM/WM remodeling and neurogenesis in both the ipsilateral and contralateral hemispheres in ABCA1^−B/−B^ stroke mice. Similar benefits were also found by others, where intraventricular infusion of ApoE improved global ischemia-induced acute brain injury in ApoE-/- mice [28]. In addition, ApoE2 administration promotes dendritic-remodeling and neurogenesis/synaptogenesis in an Alzheimer’s disease animal model [18,20,22]. In aged targeted replacement mice expressing human ApoE, ApoE2/4 synaptic terminals demonstrated the highest level of ApoE and the lowest level of Aβ compared with ApoE3/3 and ApoE4/4 lines. In ApoE2/4 terminals, the pattern of immunolabeling for ApoE and Aβ closely resembled the pattern in human control cases, and elevated ApoE was accompanied by elevated free cholesterol in ApoE2/4 synaptic terminals. These results suggest that optimal lipidation of ApoE2, compared to E3 and E4, contributes to Aβ clearance and synaptic function [20].

The brain contains the highest level of HDL in the body. Almost all brain HDL cholesterol is formed by de novo synthesis due to efficient blockade in the uptake of circulating cholesterol by the blood-brain barrier [29,30]. Different cell types in the brain have different functions in the regulation of cholesterol homeostasis, with astrocytes producing and releasing ApoE and lipoproteins, and neurons metabolizing cholesterol to 24(S)-hydroxycholesterol [31]. ApoE transports cholesterol and lipid substrates from HDL particles for maintenance of neuronal membrane and synaptic integrity in normal and injury-induced synaptic remodeling [32,33]. Thus, the availability of HDL appears to limit synapse development and axonal growth. ABCA1 mutation in humans results in Tangier Disease, which is characterized by low levels of ApoE and HDL in both the brain and plasma. Tangier disease and ABCA1-deficient patients also have an increased risk of vascular disease [10,34]. In animals, deletion of ABCA1 or ApoE, which reduces the availability of HDL cholesterol, is associated with neurodegenerative conditions [3,9,14,15,17,18,26,35], whereas upregulation of ABCA1 or ApoE enhances the processes of brain repair and remodeling and improves neurological functional outcomes [8,16,34]. 

In the present study, to investigate the mechanism underlying ApoE2-induced GM/WM remodeling and neurological benefits, we measured the brain levels of ApoE and HDL and functional outcomes in both ABCA1^fl/fl^ and ABCA1^−B/−B^ mice. To exclude estrogen’s neuroprotective effect in young adult female mice, we only examined the neurological benefit of ApoE2 in male stroke mice. We found that although ApoE2 administration increased brain levels of ApoE and HDL in ABCA1^fl/fl^ stroke mice, significant improvement in functional outcome was not evident. However, ApoE2 administration in ABCA1^−B/−B^ stroke mice significantly increased brain ApoE and HDL levels and improved neurological function, which indicates that supplementation of ApoE2 reverses brain ABCA1 deficiency-induced neurological deficits after stroke.

In adult brains, astrocytes provide the main source of cholesterol, which is taken up by neurons primarily via the ABCA1/ApoE/ApoER pathway [36]. Despite evidence from previous animal studies suggesting that ABCA1 affects ApoE levels in the brain [10,37], there is no experimental support that administration of downstream ApoE attenuates the adverse effects of brain ABCA1 deficiency via ApoER. In the present study, using ABCA1^−B/−B^ mice characterized by low levels of brain ABCA1/ApoE/HDL cholesterol, we found that intracerebral administration of ApoE2 significantly increased ApoER2 levels in the brain of ABCA1^−B/−B^ mice as well as in the PCNs and OPCs derived from ABCA1^−B/−B^ mice. In vitro studies showed that ApoE2 treatment increased cholesterol uptake by neurons and promoted neurite and axonal outgrowth in ABCA1-deficient PCNs. ApoE2 also increased proliferation and survival in ABCA1-deficient OPCs. Our results are consistent with other publications, showing that ApoE-containing lipoproteins protect neurons from apoptosis and promote neurite outgrowth and myelination in healthy adult human brains and in cultured adult mouse cortical neurons [2,21,38]. Treatment of the ApoE-/- neurons with purified human ApoE2 significantly increased neurite length more than ApoE3 [20,21]. ApoE also plays an important role in synaptic plasticity. Long-term potentiation (LTP) expression in the area of hippocampus is reduced in young ApoE-/- mice compared to age-matched control mice [39]. At the electrophysiological level, using LTP to analyze whether ApoE2 improves axonal function would complement the current cellular data, and further electrophysiological studies are warranted.

## 4. Materials and Methods

### 4.1. Animal Stroke Model

For our in vivo studies, the use of animals and experimental procedures were approved by the Institutional Animal Care and Use Committee of Henry Ford Health System and in accordance with the Institutional Animal Care and Use Committee, National Institutes of Health and ARRIVE guidelines. Adult male ABCA1^fl/fl^ (total 18) and ABCA1^−B/−B^ (total 40) mice (aged 6–7 months) were used for the in vivo study. All mice were subjected to permanent dMCAo using an electro-coagulation method (VIO 100C-Electrosurgery generator, ERBE USA, Inc., Marietta, GA, USA) [40]. At 24 h after dMCAo, animals were randomly divided into 2 groups of each strain by a non-team member using the method of drawing different colored balls. Mice were intraventricularly infused with artificial mouse CSF (100 µL; Tocris Bioscience, Bristol, UK,) or with recombinant human ApoE2 (25 µg in 100 µL artificial mouse CSF; Sigma-Aldrich Corp. St. Louis, MO, USA) by transplanting a micro-osmotic pump (D1002, 0.25 µL/h; Alzet, Cupertino, CA, USA) into the right lateral ventricle for 14 days.

ApoE levels in human and mouse CSF are similar between 6 and 12 µg/mL [10,14,22,32,37,41,42], and the physiological average ApoE level in CSF is 9.09 with a standard deviation (SD) of 2.70 µg/mL in humans [42]. Our previous data showed the ApoE level in the CSF is reduced by 40–50% in ABCA1^−B/−B^ mice (3.79 ± 0.56 µg/mL) compared with ABCA1^fl/fl^ mice (6.63 ± 0.72 µg/mL). Therefore, in the present study, in order to obtain the same normal level of ApoE in ABCA1^−B/−B^ mice as in wild-type mice, 0.25 µL/h (equal to 1.5 µg/day), a total of 25 µg ApoE2 was infused into the brain extracellular space.

### 4.2. Experimental Groups

These animals were randomly separated into three sets 24 h after dMCAo. The first set of animals (18 ABCA1^fl/fl^ stroke mice and 18 ABCA1^−B/−B^ stroke mice, *n* = 9/group) were employed for measurement of CSF ApoE and HDL levels, lesion volume, immunostaining, and functional outcome. All animals received BrdU intraperitoneally (i.p.) 50 mg/kg to label newborn cells once per day for the first 7 days. The number of animals employed in vivo was determined a priori by power calculation. dMCAo induced a consistent lesion volume and very low mortality [40,43]; therefore, in this study, 9 animals per group provided 80% power at a significance level of <0.05, assuming 20% difference in both mean and SD at the 95% confidence level and a 2-sided test. The second set of animals (total 10 ABCA1^−B/−B^ stroke mice, *n* = 5/group) were used for Golgi-staining and for measurement of dendritic branches/spines. The third set of animals (total 12 ABCA1^−B/−B^ stroke mice, *n* = 6/group) were used for WB and real-time RT-PCR assay.

### 4.3. Functional Test

The adhesive removal test, a sensitive functional test for the dMCAo model [43], was performed to evaluate the functional outcome prior to dMCAo and at days 1 (baseline before treatment), 3, 7, and 14 after dMCAo. The behavior tests were performed by an investigator blinded to the animal groups.

### 4.4. CSF Sampling and Laboratory Investigations

The CSF was drawn from the cisterna magna of each mouse 14 days after dMCAo. The levels of ApoE and HDL were measured in 2 µL of CSF, and triple tests were performed on each sample using ApoE or HDL ELISA kits.

### 4.5. Lesion Volume Measurement

All mice were euthanized 14 days after dMCAo. The animal brains were fixed by transcardial perfusion with saline, followed by perfusion and immersion in 4% paraformaldehyde, and then the brains were embedded in paraffin for slide cutting. Using a mouse brain matrix (Activational Systems Inc., Warren, MI, USA), the cerebral tissues were cut into seven equally-spaced (1 mm) coronal blocks. For lesion volume measurement, a series of adjacent 6-μm-thick sections were cut from each block and stained with hematoxylin and eosin. All stained sections representing the entire distal territory of the MCA were subjected to phase contrast microscopy to visualize the infarcted area. Infarct volume was determined with a micro-computer imaging device (MCID) imaging analysis system (Imaging Research, ST. Catharines, ON, Canada). At 14 days after ischemia, non-injured, scar, and contralateral tissue volumes were measured. The indirect lesion area, in which the intact area of the ipsilateral hemisphere was subtracted from the area of the contralateral hemisphere, was calculated [44]. Lesion volume is presented as the actual indirect lesion volumes [8].

### 4.6. Histochemical and Immunohistochemical Staining Assessment

For histochemical or immunohistochemical staining, every 10th coronal 6 μm section was cut from the center of the lesion (bregma −1 mm to +1 mm), and a total of 5 paraffin sections were used. Histochemical staining for BS**^+^** and LFB**^+^**, and immunohisto-staining for Syn**^+^** (Chemicon, Temecula, CA, USA) and SMI31 (Covance, Princeton, NJ, USA) single staining were performed. Double-immunohisto staining for nestin (Santa Cruz, Santa Cruz, CA, USA) and Sox2 (Santa Cruz), and 4’,6-diamido-2-phenylindole (DAPI, Santa Cruz) were employed.

The Golgi-Cox impregnation method along with the FD Rapid Golgi Stain kit (FD Neuro-Technologies, Columbia, MD, USA) were used to identify Golgi-Cox impregnated dendrites and spines, according to kit instructions [45].

### 4.7. Image Acquisition and Quantification for Immunostaining Analysis

The images were acquired and measured in both the ischemic boundary zone (IBZ, defined as the area surrounding the lesion which morphologically differs from the surrounding normal tissue), in the ipsilateral (Figure 2A, squares 1–8) and matched contralateral regions (Figure 2A, within the outlined areas) using MCID. Images that were acquired from a total of five slides from each brain with each slide containing 4 fields of view. For Syn^+^ measurement, images from the cortex (squares 1–4) were acquired; for BS^+^/LFB^+^/SMI31^+^ measurement, the images from the CC (squares 5–8) were acquired. The densities of Syn^+^/BS^+^/LFB^+^/SMI31^+^ area were calculated.

For Golgi staining, dendritic arborization was measured under a 40× objective and 10 intact neurons from layer III of the cortex were chosen (squares 2–3), and the number of dendritic branches including primary and secondary and the dendritic length were counted. For evaluation of spine density, 10 neurons from each brain sample in layer III of the cortex were digitized under an oil immersion 100× objective, and spine numbers in 10 stretches of secondary dendrites of at least 10 µm in length were analyzed, as previously described [46].

For measurement of neurogenesis, 40× images from the SVZ (square 9) for nestin^+^ and Sox2^+^ [47,48] were acquired. The total number of nestin^+^/Sox2^+^ cells and the percentage of nestin^+^/Sox2^+^ double with BrdU^+^ cells of the total number of nestin^+^/Sox2^+^ cells in the SVZ were quantified.

### 4.8. Real-Time RT-PCR and WB Assay

The ipsilateral ischemic brain tissue and the homologous contralateral brain tissue (Figure 2A) were isolated. Total RNA was isolated and quantitative-PCR was performed in the ABI Prism 7000 sequence detection system (Thermo Fisher Scientific, Carlsbad, CA, USA), using the Quantitec SYBY Green PCR kit (Qiagen, Germantown, MD, USA). The following primers were designed using Primer Express software (ABI, Thermo Fisher Scientific, Carlsbad, CA, USA). GAPDH: Fwd, AGAACATCATCCCTGCATCC; Rev: CACATTGGGGGTAGGAACAC. MBP: Fwd, ATCCAAGTACCTGGCCACAG; Rev, CCTGTCACCGCTAAAGAAGC; ApoER2: Fwd, AGTGTCCCGATGGCTCTGAC; Rev, CAGCTTAACTTCTCGGCAGGA.

Equal amounts of brain tissue lysate were subjected to WB analysis. Specific proteins were visualized using a SuperSignal West Pico chemiluminescence kit (Pierce, Appleton, WI, USA). The following primary antibodies were used: anti-ApoER2 (1:1000, ab204112, Abcam, Cambridge, UK), anti-Syn (1:5000, MAB5258, Chemicon, Temecula, CA, USA), anti-MBP (1:500, MAB386, Millipore, Burlington, MA, USA), and anti-β-actin (1:10000; ab6276, Abcam, Cambridge, UK).

### 4.9. PCN Culture, ApoR2 Expression, and Cholesterol Distribution Analysis

The PCNs were isolated from E17-18 C57BL/6, ABCA1^fl/fl^, and ABCA1^−B/−B^ embryos and were cultured as previously described [49]. The PCN culture was a mixed culture of cortical neurons with astrocytes (we did not kill astrocytes in the cultures). This culture contained some ApoE in the medium secreted from astrocytes. In order to demonstrate whether PCN expresses ApoER2 and to test whether ApoE2 treatment increases cholesterol transport from outside into neurons, 4 μg/mL ApoE2 was chosen.

Briefly, the C57BL/6-PCNs on day-in-vitro 4 (DIV4) were used and treated with ApoE2 or dimethyl sulfoxide (DMSO, vehicle control) for 48 h. ApoER2 with fluorescein isothiocyanate (FITC, Thermo Fisher Scientific) and TUJ-1 (a phenotypic marker of neural cells, 1:1000, Covance, Princeton, NJ, USA) with Cy3 double immunofluorescent staining was performed, and 40× magnification images were acquired. The intracellular free cholesterol particles were monitored by Filipin III staining using a cell-based cholesterol assay kit (ab133116, Abcam, Cambridge, UK). Briefly, on DIV6, the culture medium was removed and the PCNs were fixed with fixative solution for 10 min. The PCNs were then washed with cholesterol detection wash buffer, 3 × 5 min each. Then, 100 μL of Filipin III solution was added to each well and incubated in the dark for 30–60 min. The cells were washed with wash buffer, 2 × 5 min each. The staining was examined using a fluorescent microscope at an excitation of 340–380 nm and emission of 385–470 nm. Pictures were taken immediately because Filipin fluorescent staining is rapidly photobleached.

### 4.10. PCN Neurite and Axonal Outgrowth Measurements

To test whether ABCA1^−B/−B^ decreases neurite outgrowth and whether ApoE2 dose-dependently increases neurite outgrowth after ischemia, the ABCA1^fl/fl^-PCNs and ABCA1^−B/−B^-PCNs were subjected to 2 h of oxygen and glucose deprivation (OGD) on DIV4 followed by 24 h of reperfusion. The DIV5 OGD-PCNs were randomly divided into 5 groups (*n* = 6 wells/group): (1) non-treatment ABCA1^fl/fl^-PCNs; (2) non-treatment ABCA1^−B/−B^-PCNs; (3) ABCA1^−B/−B^-PCNs + ApoE2 2 µg/mL; (4) ABCA1^−B/−B^-PCNs + ApoE2 4 µg/mL; and (5) ABCA1^−B/−B^-PCNs + ApoE2 8 µg/mL for 24 h. Based on the above discussion about ApoE levels in the CSF of ABCA1^−B/−B^ mice, we chose low (2 μg/mL), intermediate (4 μg/mL), and high (8 μg/mL) ApoE2 doses to obtain the normal wild-type CSF level (ranging from 3.79 + 2, 3.79 + 4, and 3.79 + 8 μg/mL), respectively. The PCN cultures were then stained with TUJ1 and photographed using a 10× objective fluorescent microscope (Zeiss, Oberkochen, Germany). The average length of the 20 longest neurites in each well was calculated [49].

For testing whether ABCA1^−B/−B^ decreases axonal outgrowth and whether ApoE2 attenuates ABCA1^−B/−B^-induced reduction of axonal outgrowth, the ABCA1^fl/fl^–PCNs and ABCA1^−B/−B^-PCNs were cultured in microfluidic chambers (Standard Neuron Device; catalog No SND450, Xona Microfluidics, Temecula, CA, USA) [49]. On DIV4, the PCN cultures were divided into same groups and treated the same as in the above neurite outgrowth measurements. All axon cultures were allowed to grow for an additional 48 h, and the axonal outgrowth was measured on DIV6. The average length of the 10 longest axons in each well was calculated.

### 4.11. Primary OPC Culture and Purity Analysis

The cerebral cortex was dissociated from 2-day-old post-natal C57BL/6 mice. The isolated cells were cultured and purified as previously described [50]. Briefly, cells were replanted in poly-D lysine coated 75 cm^2^ flasks, and maintained in Dulbecco’s Modified Eagle’s Medium (DMEM) medium containing 20% horse serum and 1% penicillin/streptomycin for 10 days. The flasks were shaken overnight on an orbital shaker at 200 rpm for 1 h to remove microglia then were shaken overnight. The medium was collected and plated on non-coated tissue culture dishes for 1 h at 37 °C and 5% CO_2_ to eliminate contaminating astrocytes and microglia. The non-adherent cells were collected and suspended with OPC growth serum-free medium (DMEM, 1% insulin, transferrin and sodium selenite (ITS) liquid media supplement, 10 ng/mL platelet-derived growth factor AA, 10 ng/mL basic fibroblast growth factor), and re-plated in a 96-well plate coated with Poly-DL-Ornithine (10μg/mL in PBS, P0421-100MG, Sigma-Aldrich Corp. St. Louis, MO USA).

To determine the OPC purity on DIV4, the adherent C57BL/6-cells were immunofluorescent stained with platelet-derived growth factor receptor alpha (PDGFRα) and O4 (marker of OPCs), and DAPI (nuclei marker). The number of PDGFRα/O4/DAPI positive cells were counted. Our data indicated more than 90% of cells WERE OPCs at DIV4 (Figure 8A).

### 4.12. OPC Proliferation and Death Measurement 

To examine whether ABCA1^−B/−B^ decreases OPC proliferation and survival and whether ApoE2 attenuates ABCA1^−B/−B^-induced reduction in OPC proliferation and survival, the ABCA1^fl/fl^–OPCs and ABCA1^−B/−B^-OPCs were used, and randomly divided into 5 groups: (1) non-treatment ABCA1^fl/fl^–OPCs; (2) non-treatment ABCA1^−B/−B^-OPCs; (3) ABCA1^−B/−B^-OPCs + ApoE2 2 µg/mL; (4) ABCA1^−B/−B^-OPCs + ApoE2 4 µg/mL; and (5) ABCA1^−B/−B^-OPCs + ApoE2 8 µg/mL.

For OPC proliferation measurements, 5000 purified OPC cells were plated in 96-well plates coated with Poly-DL-Ornithine hydrobromide (Sigma-Aldrich Corp. St. Louis, MO, USA) and incubated at 37°C/5% CO_2_, and cultured with or without ApoE2 treatment for 3 days. Then, 20 µL 3-[4,5-dimethylthiazol-2-yl]-5-[3-carboxymethoxyphenyl]-2-[4-sulfophenyl]-2H-tetrazolium (MTS) was added to each well and the MTS assay (a cell proliferation assay) was performed 3 h after treatment using the CellTiter 96 Aqueous One Solution Cell Proliferation Assay kit (Promega, Madison, WI, USA). Data are presented with absorbance (OD value) recorded at 490 nm.

For OPC survival measurement, the OPCs were allowed to grow for 24 h with or without ApoE2 treatment, and then the lactate dehydrogenase (LDH) assay (a cell death assay) was performed as previously described [49]. The LDH levels in the media (secreted) and the total LDH level in both the media and cells were measured using the CytoTox 96 non-radioactive cytotoxicity assay kit (Roche, Indianapolis, IN, USA) following the manufacture’s protocol. Data are presented as percentage of LDH level in the media to total LDH.

### 4.13. Statistical Analysis

Two-way analysis of covariance (2-way ANOVA) followed by Tukey’s post hoc test were performed for analysis of: (1) ApoE/HDL levels, lesion volume, and ApoER2 level in PCNs/OPCs for comparison of gene deficient (ABCA1^−B/−B^ versus ABCA1^fl/fl^) and treatment effect (with versus without ApoE2), respectively; (2) measurement of gene/protein expression for comparison of region (ipsilateral versus contralateral) and treatment effect (with versus without ApoE2). Three-way ANOVA with repeated measure was used for functional analysis. Independent 2-sample *t*-test was used for comparison difference of Syn/BS/SMI31/LFB/nestin/Sox2 immunostaining and Golgi staining between CSF administered ABCA1^−B/−B^ stroke mice and ApoE2 administered ABCA1^−B/−B^ stroke mice. One-way ANOVA was used for analysis in vitro cell culture studies. Data are presented as mean ± standard error (SE), and a value of *p* < 0.05 was considered significant.

## 5. Conclusions

This study was a proof of concept and mechanism experimental study and not preclinical research. We are the first to demonstrate that intracerebral supplementation of ApoE2, the downstream molecule of ABCA1 in brain, compensates for the neurological deficits of brain ABCA1 deficiency in both an experimental stroke model and cell cultures, at least partially by increasing brain ApoER2 and cholesterol uptake.
